# Retrospective analysis of earthquake related crush injurie patients in ICU: 6-February earthquake in Türkiye

**DOI:** 10.1007/s00068-025-02771-4

**Published:** 2025-02-21

**Authors:** Sahin Temel, Recep Civan Yuksel, Ahmet Safa Kaynar, Mustafa Caliskan, Berna Demir, Mustafa Alkan, Birkan Ulger, Kamil Deveci, Hilal Sipahioglu, Hatice Metin, Selda Kayaalti, Elif Kaya, Canan Baran Unal, Aliye Esmaoglu, Murat Sungur, Dincer Goksuluk, Kursat Gundogan

**Affiliations:** 1 Division of Medical Intensive Care, Department of Medicine, School of Medicine, Erciyes University, 38039. Melikgazi, Kayseri, Turkey; 2 Intensive Care Unit, Kayseri City Hospital, Ministry of Health, Kayseri, Turkey; 3 Division of Anesthesiology and Reanimation Intensive Care, School of Medicine, Erciyes University, Kayseri, Turkey; 4 Department of Clinical Nutrition, Erciyes University Health Sciences Institute, Kayseri, Turkey; 5 Department of Biostatistics, School of Medicine, Erciyes University, 38030 Kayseri, Turkey

**Keywords:** Crush injurie, Earthquake, ICU, Kahramanmaras, Türkiye

## Abstract

**Background:**

On February 6, 2023 a devastating earthquake hit the south-eastern region of Türkiye and thousands of people were either injured or died. The aim was to determine the characteristics, treatment and clinical outcomes of critically ill patients with crush injuries in ICU.

**Subjects/methods:**

This study was performed with a retrospective design in ICU. Patients were included as follows; effected 6 February earthquake, need ICU treatment and being crush syndrome.

**Results:**

A total of 62 patients were included. The mean age was 41 ± 19 years and 47% were male. The median APACHE II score was 14. The most common ICU admission was multitrauma and crush injury. A total of %77 patients were needed surgical procedure (most of them extremities surgery (36%)and fasciotomy 36% due to compartment syndrome) and %24 patients had extremity amputation. AKI was developed in %65 of patients. A total of 26 (%40) patients were received RRT. The mNUTRIC score (*p* = 0.022), the BUN (*p* = 0.043) and the blood lactate level (*p* = 0.012) were identified as independent risk factors for 28-day mortality. An independent risk factor for limb amputation was identified in patients with high APACHE II and SOFA scores (*p* = 0.026, *p* = 0.034, respectively). The 28-day mortality was 13%.

**Conclusions:**

As a result of the study, most of the patients need surgical operations and a quarter of patients required extremity amputation. AKI developed at a high rate and 40% of those patients needed RRT. The mNUTRIC score was found to be the most powerful predictor of mortality at 28 days.

**Supplementary Information:**

The online version contains supplementary material available at 10.1007/s00068-025-02771-4.

## Introduction

Every year, millions of people around the world experience primary disasters, which can either be caused by nature or humans [1]. Natural disasters include earthquakes, cyclones, hurricanes, flooding, and landslides. While man-made disasters consist of terrorist attacks, Aeroplan or train crashes and wars [2]. Approximately 800 million people currently reside in areas that are prone to earthquakes or severe tropical cyclones [3]. The earthquake that occurred on 6th February 2023 and impacted Türkiye and Syria has led to more than 50,000 fatalities and displaced millions of people. Major earthquakes with high magnitude on the Richter scale have the potential to cause significant harm and loss of life, primarily due to multiple injuries from major trauma and entrapment [4]. There are higher chances of crush injuries and crush syndrome during such incidents [5].

Crush injuries occur as a result of direct physical trauma or compression to the part of the human body, particularly the lower extremities [6]. This can lead to various complications such as asphyxia, severe orthopedic injury, compartment syndrome, hypotension, and organ damage, including acute kidney injury [7, 8]. Crush syndrome is a condition that arises from a significant and traumatizing muscle injury, affecting the entire body systemically [6, 7].

Data from previous disasters indicate that around 80% of trapped victims die immediately due to severe injuries. About 10% of victims get crush injuries and 10% receive minor injuries [9].

In this study, we aimed to analyze the laboratory characteristics, treatment and clinical outcomes of critically crush injured patients admitted to the ICU at the time of their initial admission and follow-ups.

## Materials and methods

This was a retrospective study performed in one of the hospital ICUs in Turkey. Ethics approval for the study was obtained from the Ethics Committee of Erciyes University (**number: 2023/679**,** dated:25.10.2023**). Since the study design was retrospective, consent for publishing the patient data was not obtained from the patients. The study included all patients who sustained crush injuries and subsequently required management in the ICU.

**Fig. 1:**
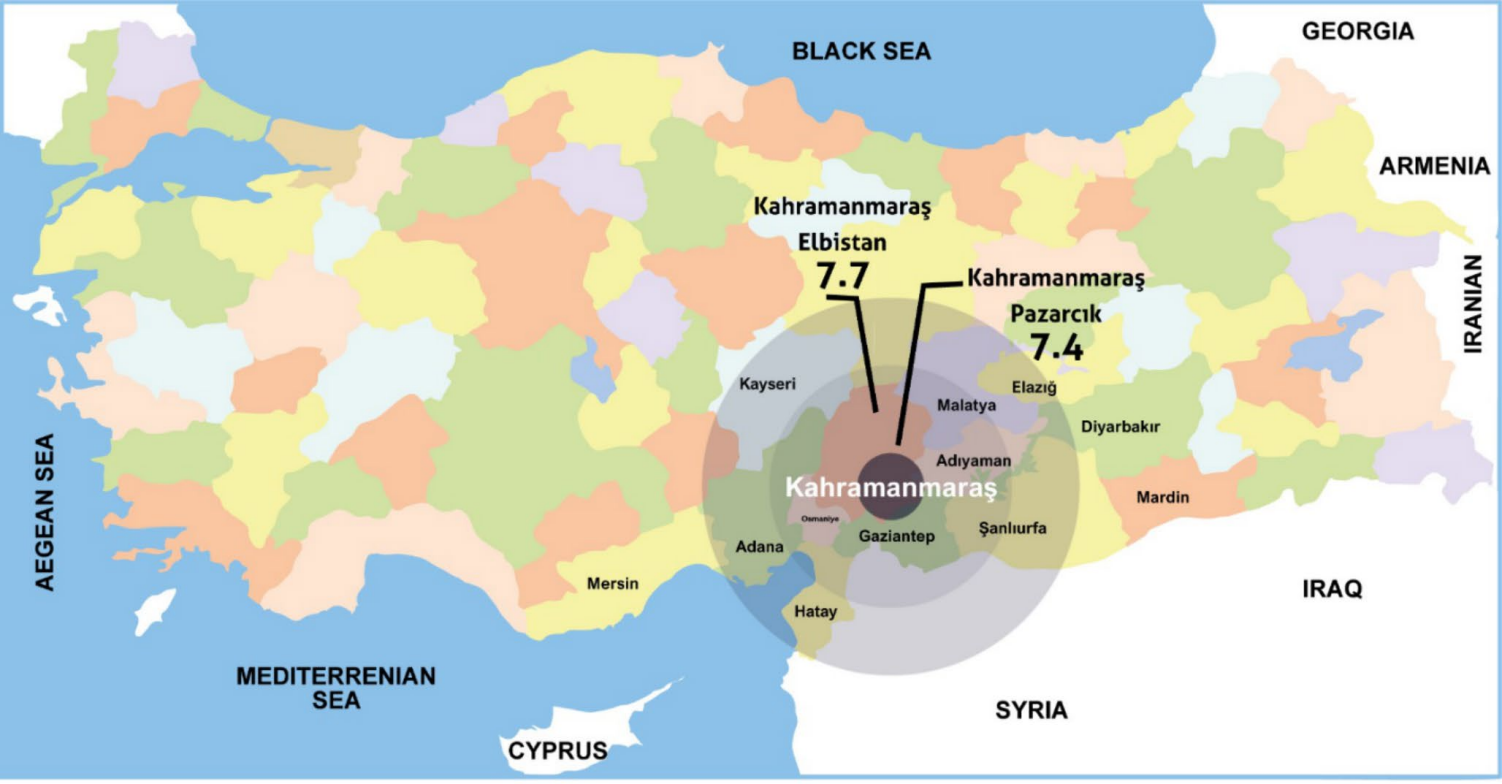
Earthquake map of Türkiye

The city of Kayseri is one of the areas that were moderately affected by the earthquake on February 6, 2023. The earthquake caused material damage in Kayseri, but did not result in any injuries or deaths. Hospital buildings and other official buildings remained intact. Two major hospitals in the city of Kayseri, City Hospital and Erciyes University Hospital, accepted earthquake victims who needed outpatient, inpatient, and intensive care from the earthquake-stricken area (Fig. 1).

### Patients’ data

All the data were recorded from patients’ charts and the hospital electronic medical system. The treatments received and clinical results were recorded during the first 10 days of the patient’s stay in the ICU.

#### Intensive care unit admission and follow-up days’ parameters

Demographic information such as age, gender, the region where the earthquake occurred, and the date they arrived at the ICU were recorded. The reasons for admission to the ICU of patients (crush injury, single or multiple trauma), accompanying underlying diseases, Glasgow coma score (GCS), APACHE II score, SOFA score, modified NUTRIC score [10] were also recorded. The oxygen support needed by patients is classified as follows: not receiving, nasal/mask, high flow oxygen support, non-invasive mechanical ventilation, and invasive mechanical ventilation (IMV). If patients receive IMV support, the parameters of the mechanical ventilator are recorded. The whole blood count and blood biochemistry values of patients including creatinine (Cr), creatine kinase (CK), myoglobin, C-Reactive Protein (CRP), coagulation parameters, blood gas analysis, and urine analysis results were recorded. Antibiotics, vasopressors, proton pump inhibitors, and deep vein thrombosis prophylaxis medications were recorded for patients in the ICU. In addition, fluid therapy and their quantities applied for the treatment of crush injuries in patients, sodium bicarbonate (HCO3), mannitol, blood products, fluid balance, nutritional and renal replacement therapies (RRT) were recorded. The patients’ direct chest X-rays, extremity bone X-rays, and computed tomography (CT) were evaluated. Finally, surgical procedures or operative procedures performed in the emergency department or on the first and follow-up days of the ICU were examined. AKI was defined KDIGO criteria [11].

### Statistical analysis

Statistical analyses were conducted using R (version 4.3.2) and IBM SPSS Statistics (version 26). Numerical variables were summarized as means ± standard deviations or medians with ranges, based on normality assessed through graphical (Q-Q plots, histograms) and analytical methods (Shapiro-Wilk test). Categorical variables were described as frequencies and percentages. Comparisons between groups employed Student’s t-test for normally distributed variables and Mann-Whitney U test for non-normal distributions. Survival analyses included univariate Cox or logistic regression models for clinical outcomes, with predictors showing *p* < 0.20 included in multivariate models, and results expressed as hazard or odds ratios with 95% confidence intervals. Kaplan-Meier analysis with log-rank tests compared survival curves. Statistical significance was set at *p* < 0.05.

## Results

A total of 62 patients were enrolled in this study. The median age was 33 years (range:18–84) and 47% were male. More than half (51 patients, 82%) of the patients were admitted to the ICU for multiple trauma and crush injuries. The 28-day mortality rate was 13%. The first day median APACHE II score was 14 (min-max: 1–41) and APACHE II score was higher in those patients who died during the treatment as compared to those who survived (*p* = 0.009). When analyzing the NUTRIC score, which assesses the risk of malnutrition in patients with crush injury, the median mNUTRIC score was 2(0–7) and was higher in patients who died (*p* = 0.003). The median SOFA score was 4 (0–14) and it was significantly higher in the non-survivor patient group (*p* = 0.008). (*p* = 0.008). Among the patients with crush injuries 71% developed acute kidney injury (AKI). Of whom 26 were managed with renal replacement therapy (RRT); 22 (%) patients received intermittent hemodialysis, and four patients received continue venovenous hemodiafiltration (CVVHDF)). Subcutaneous heparin 32%, low-molecular-weight heparin 34%, and pneumatic compression stockings 34% were used to treat DVT during ICU stay. In these patients, 79% of the normal saline and 21% of the balanced crystalloid fluid resuscitation were continued in the ICU after being administered at the earthquake site and in the emergency department. Furthermore, 40 patients (65%) were initiated on HCO3, while 10 patients (16%) were commenced on mannitol. The mean total fluid intake of the patients on admission day was 5264 ± 2684 ml (min: 1000 ml - max: 15625 ml). During the ICU follow-up period, 71% of patients underwent surgery and 24% of these patients underwent limb amputation. The median length of stay in the hospital and ICU was 20 days (min-max: 1-118), 6 days (min-max:1-118) respectively. Detailed patient demographic and clinical outcomes are shown in Table 1. All patients received proton pump inhibitors (PPI) for stress ulcer bleeding prophylaxis and broad-spectrum antibiotics during intensive follow-up.

**Table 1:** Demographic and clinical characteristics of the participants upon admission and during their stay in the ICU

Variables	All patients *N* = 62	Survival *N* = 54	Non-survival (28-day mortality) *N* = 8	*p*
Age, year (median)(min-max)	33 (18–84)	30 (18–84)	62 (33–79)	**0.011**
**Gender**,** n (%)**				0.125
Male	29 (47)	23 (43)	6 (75)	
Female	33 (53)	31 (58)	2 (25)	
**Reason for ICU admission**,** n (%)**				0.142
Isolated crush injury	11	8	3	
Crush injury with associated other injuries	51	46	5	
APACHE II score, (median)(min-max)	14 (1–41)	13.5(1–41)	28 (11–34)	**0.009**
Modified NUTRIC score, (median)(min-max)	2 (0–7)	2 (0–7)	4 (1–7)	**0.003**
SOFA score, (median)(min-max)	4 (0–14)	4 (0–14)	8 (2–14)	**0.008**
**Oxygen support**,** n (%)**				0.378
Invasive Mechanical Ventilation	9	8	1	
Face mask	43	36	7	
Room air	10			
**Acute kidney injury**,** n (%)**	44 (71)	36 (67)	8 (100)	0.092
**Type of nutrition**,** n (%)**				0.705
Oral diet (R1-3)	42 (68)	37 (69)	5 (63)	
EN tube feeding	20 (32)	17 (32)	3 (38)	
**Type of renal displacement therapy**,** n (%)**				0.071
Intermittent hemodialysis (IHD)	22 (36)	20 (37)	2 (25)	
CVVHDF	4 (7)	2 (25)	2 (50)	
**Anti-Coagulant therapy**				0.831
Heparin	20 (32)	16 (30)	4 (50)	
Low molecular weight heparin	21 (34)	20 (37)	1 (13)	
Pneumatic compression socks	21 (34)	18 (33)	3 (38)	
Vasopressor drugs, n(%)	4 (7)	2 (4)	2 (25)	0.077
**Initiated fluid treatments**,** n (%)**				0.770
0.9 NaCL (normal saline)	49 (79)	42 (78)	7 (88)	
Balanced fluid (isolyte S)	13 (21)	12 (20)	1 (13)	
Treatment with bicarbonate, n(%)	40 (65)	34 (63)	6 (75)	0.700
Mannitol therapy, n (%)	10 (16)	7 (13)	3 (38)	0.111
Surgical operation, n(%)	44 (71)	36(67)	8 (100)	0.092
Organ amputation, n (%)	15 (24)	10 (19)	5 (63)	**0.016**
Length of hospital stay, day(min-max)	20 (1-118)	21 (1-118)	6 (1–26)	**< 0.001**
Length of ICU stay, day (min-max)	6 (1-118)	6 (1-118)	3.5 (1–26)	0.388

APACHE II: The Acute Physiology and Chronic Health Evaluation, IMV: Invasive Mechanical Ventilation, SOFA: Sequential Organ Failure Assessment, IHD: Intermittent Hemodialysis, CVVHDF: Continuous Venovenous Hemodiafiltration

### Laboratory data

The results of whole blood count (WBC), coagulation tests, biochemical analysis, urine analysis, and blood gas analysis of the patients are listed in Table 2. The median BUN was 31 mg/dl (range: 5.30–146). Patients who died had a BUN of 55.5 mg/dl (29–143) (*p* = 0.025). The median potassium (K), one of the serum electrolytes, was 4.80 (range:2.40–7.60). When comparing living and deceased patients, it was statistically higher in deceased patients (5.60 (4.80–7.50) (*p* = 0.031). The median CRP was 121.3 mg/dl in patients followed up for crush injury. When comparing patients who survived (116.5 mg/dl) and those who died (136.0 mg/dl), it was higher in those who died (*p* = 0.033).

**Table 2:** Laboratory characteristics of patients with crush injury upon admission to the ICU

Variables Median (min-max)	All patients *N* = 62	Survival *N* = 54	Non-survival (28-day mortality) *N* = 8	*p*
**WBC (x1000) (0^3/ µL )**	14.08 (1.7–31.02)	14 (1.7–31.02)	17.8 (8.18–30.05)	0.248
**Hemoglobin(g/dL)**	10.95 (5.9–121)	10.95 (5.9–121)	11.2 (8.1–96)	0.667
**Platelets (x1000) (10^3/µL)**	189 (81–496)	185 (81–496)	261.5 (96–333)	0.133
**BUN (mg/dL)**	31 (5.30–146)	27.7 (5.30–146)	55.5 (29–143)	**0.025**
**Creatinine (mg/dL)**	1.74 (0.3–7.40)	1.38 (0.3–7.40)	2.89 (0.93–4.24)	0.211
**CK (x1000) (µ/L)**	12.85 (0.31–234.7)	15.8 (0.31–234.7)	7.6 (0.55–183.7)	0.965
Myoglobulin* (x1000) (ng/ml)	3 (0.17–47.03)	3 (0.17–47.03)	23.2 (3–43.4)	0.348
**Sodium (mmol/L)**	137 (117–163)	136.5 (117–163)	138 (126–154)	0.494
**Potassium (mmol/L)**	4.80 (2.40–7.60)	4.55 (2.40–7.60)	5.60 (4.80–7.50)	**0.031**
**Chlorine (mmol/L)**	103 (83–122)	103 (83–122)	102.5 (94–118)	0.674
**LDH**,** µ/L**	736.5 (78.0-8004.0)	715.0 (78.0-5639.0)	1764.5 (357.0-8004.0)	0.178
**D-dimer (x1000) (µg/L)**	4.08 (0.72–32.8)	4.08 (0.72–32.8)	8.89 (5.3–20.2)	0.143
**Fibrinogen (x1000)** **(mg /dL)**	1.03 (0.08–8.7)	0.49 (0.08–8.7)	4.28 (0.02–8.25)	0.223
**INR**	1.20 (0.88–2.52)	1.14 (0.88–2.52)	1.50 (1.29–2.11)	**0.003**
**HCO3 (mmol/L)**	19 (9–29)	19 (9–29)	14.4 (9–22.4)	0.219
**Lactate (mmol/L)**	1.70 (0.49–13.50)	1.69 (0.60–6.55)	2.17 (0.49–13.50)	0.198
**Urine PH**	5.5 (5–7)	5.5 (5–7)	5.5 (5–5.5)	0.331
**Blood PH**	7.36 (6.88–7.93)	7.36 (7.15–7.51)	7.24 (6.88–7.93)	0.259
**CRP (mg/dL)**	121.3 (5.0-352.0)	116.5 (8.0-352.0)	136.0 (5.0-318.0)	**0.033**

*Myoglobulin levels were measured 31 patients. Kayseri City Hospital did not measure serum myoglobulin levels. INR: International normalized ratio, HCO3: Bicarbonate, CRP: C-reactive protein, CK: Creatinine kinase,

### Radiologic data

Table 3a shows the injuries that were detected on the radiological images. Limb fracture, pneumothorax (12 patients) and pleural effusion with hemothorax (8 patients) were the most common types of pathology detected.

**Table 3a:** The patients’ radiologic images evaluation upon admission to the ICU and emergency department

Variables	Number of patients
Chest X-ray radiograph	
Pneumothorax	12
Pleural effusion	5
Hemothorax	3
Pneumomediastinum	2
Pulmonary contusion	2
Rib fracture	2
**Bone radiography**	
Limb fracture	40
**Computed tomography scan** **(CT)**	
Bone fracture (cranium, limb, rib)	39

**Table 3b:** List of surgical procedures carried out on patients with crush injuries in the ICU

Type of operation	Number of operations
Compartment fasciotomy	18
Upper limb amputation	5
Bilateral/unilateral below-knee amputation	4
Negative pressure chest tube	4
Humerus fracture fixation	3
Acetabular fracture fixation	3
Debridement and bleeding control	1
Femur intramedullary nail operation	1
Femur corpus fixation	1
Diagnostic laparotomy	1
Finger amputation	1
Ankle fracture fixation	1
Tibia and fibula fracture surgery	1

Compartment fasciotomy, upper extremity amputation, below-knee amputation, and negative pressure chest tube were the most common surgical procedures performed in the ICU (Table 3b).

**Fig. 2:**
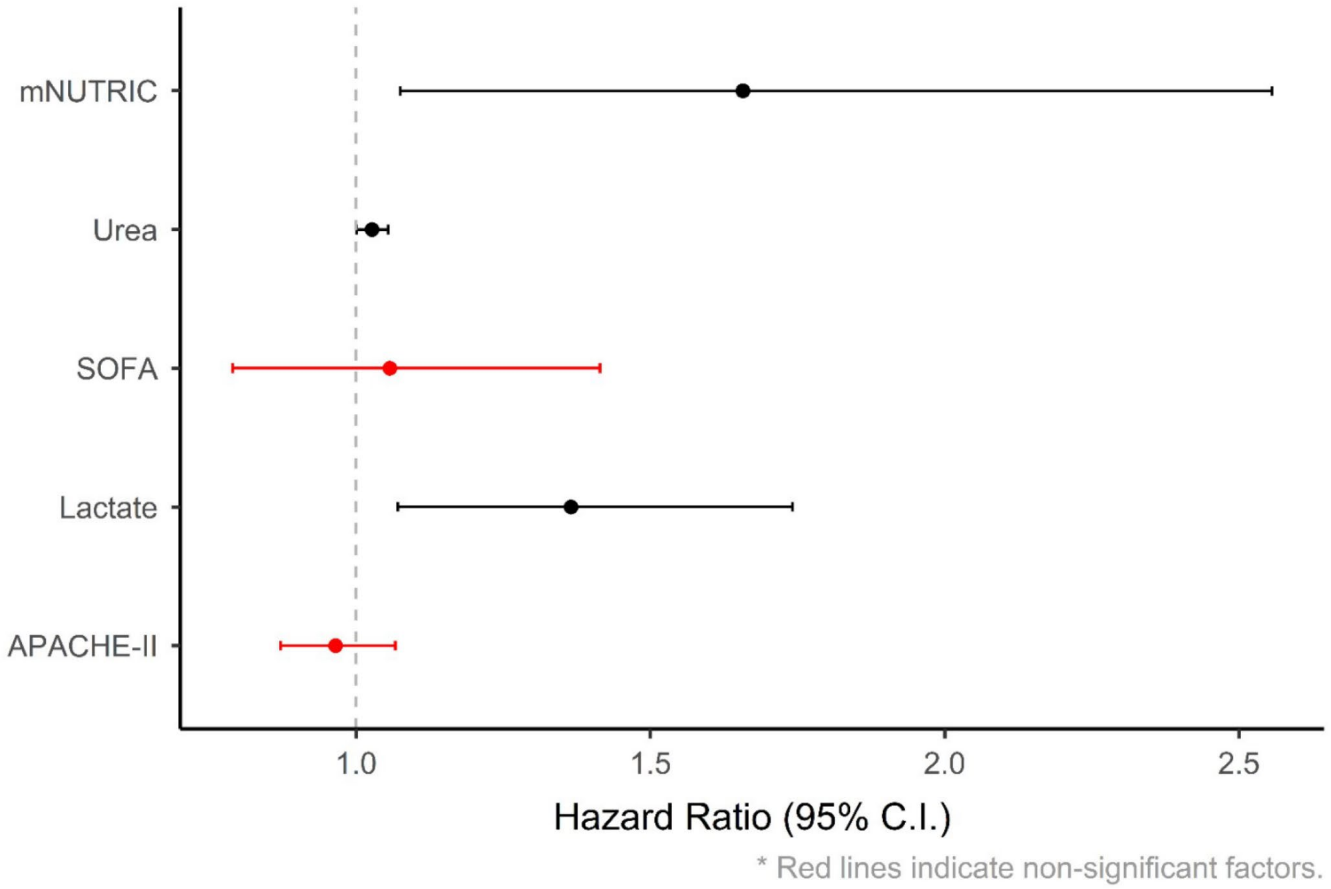
Cox regression analysis was used to identify risk factors for 28-day mortality in patients with a crush injury

**Fig. 3:**
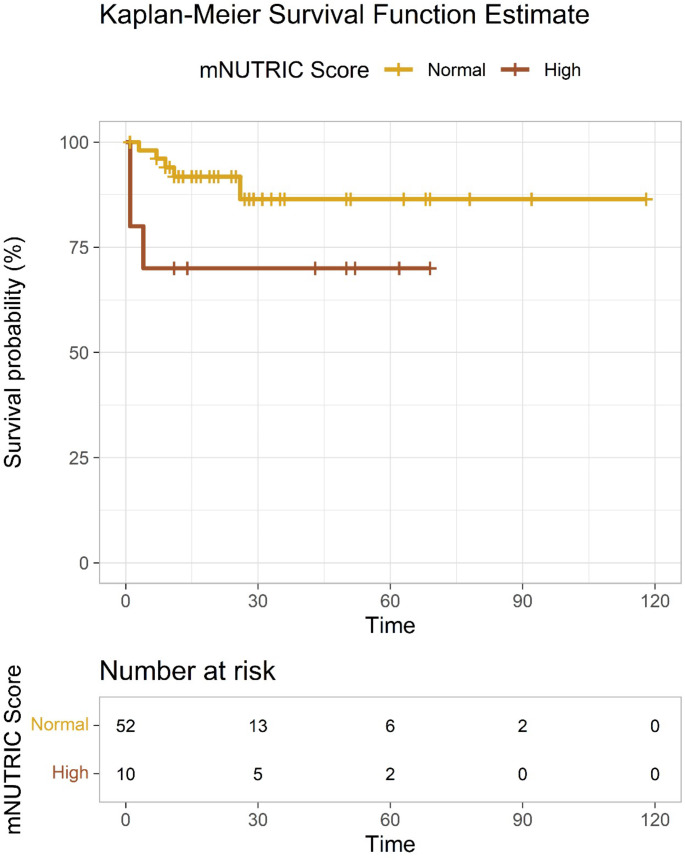
Kaplan-Meier survival curves according to the mNUTRIC score

Independent risk factors for 28-day mortality in patients with crush injury were identified using Cox regression analysis. The mNUTRIC score (Exp. B: 1.657), the BUN (Exp. B: 1.027) and the blood lactate level (Exp. B: 1.365) were determined as independent risk factors for 28-day mortality (Table 4a) (Fig. 2). mNUTRIC score among the survivors and non-survivors is shown in Fig. 3 (*p* = 0.022).

**Table 4a:** Independent risk factors for 28-day mortality in patients with crush injury were identified using Cox regression analysis

	B	SE	Wald	df	Sig.	Exp(B)	95,0% CI for Exp(B)	
							Lower	Upper
APACHE II score	-0.036	0.052	0.487	1	0.485	0.965	0.872	1.067
mNUTRIC score	0.505	0.221	5.241	1	**0.022**	1.657	1.075	2.555
BUN	0.027	0.013	4.096	1	**0.043**	1.027	1.001	1.054
Lactate	0.311	0.124	6.284	1	**0.012**	1.365	1.070	1.741
SOFA score	0.055	0.148	0.140	1	0.709	1.057	0.790	1.414

**Table 4b:** Univariate logistic regression analysis of independent risk factors for organ amputation in patients with crush injury

	B	S.E.	Wald	df	Sig.	Exp (B)	95% CI for EXP(B)	
							Lower	Upper
APACHE II score	0.071	0.032	4.980	1	**0.026**	1.073	1.009	1.142
SOFA score	0.208	0.098	4.473	1	**0.034**	1.231	1.015	1.492

APACHE II: SOFA:

**Table 4c:** Univariate logistic regression analysis of independent risk factors for mNUTRIC score in patients with acute kidney injury

	B	S.E.	Wald	df	Sig.	Exp (B)	95% CI for EXP(B)	
							Lower	Upper
mNUTRIC score	0.229	0.163	1.971	1	0.160	1.258	0.913	1.732

Univariate logistic regression analysis of independent risk factors for limb amputation in crush injury patients is shown in Table 4b. Independent risk factor for limb amputation identified in high APACHE II and SOFA scores (*p* = 0.026, *p* = 0.034 respectively). When patients with AKI were assessed for the development of malnutrition, malnutrition was 1.32-fold higher in patients with AKI. Although this was not statistically significant (*p* = 0.160) (Table 4c).

Serum creatinine, CK, and myoglobulin levels were compared between patients with and without AKI during the 10-day follow-up period. Serum creatinine levels clinically increased in patients with AKI (Fig. 4a) (Supplemental Table 1). Serum CK and myoglobulin levels decreased in patients with AKI (Fig. 4a and b) (Supplemental Tables 2 and 3).

**Fig. 4a:**
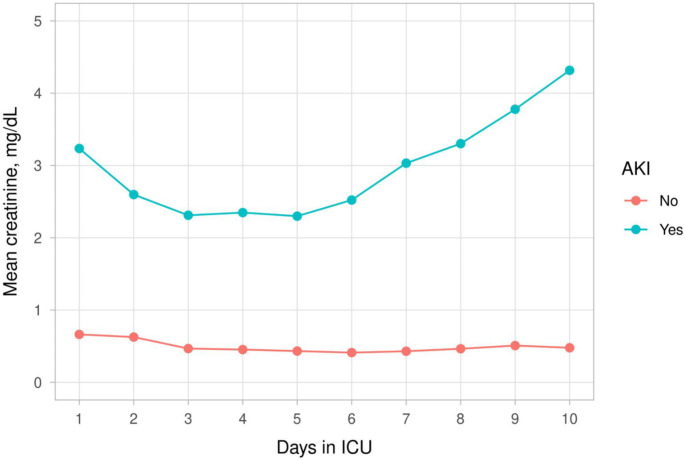
Mean serum creatinine levels - first 10 days of follow-up with and without AKI

**Fig. 4b:**
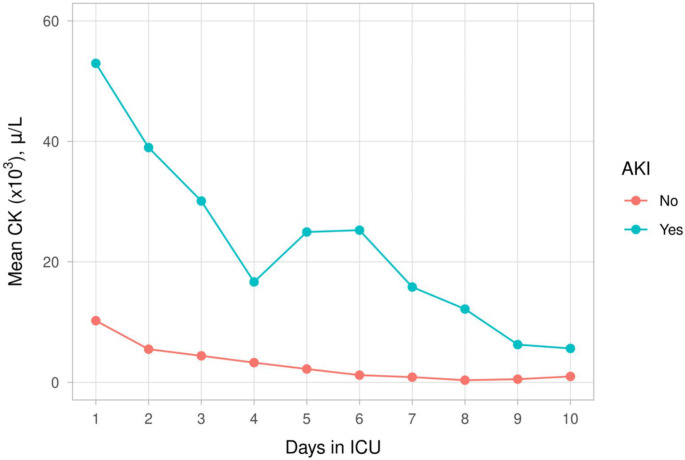
Mean serum CK levels - first 10 days of follow-up with and without AKI

**Fig. 4c:**
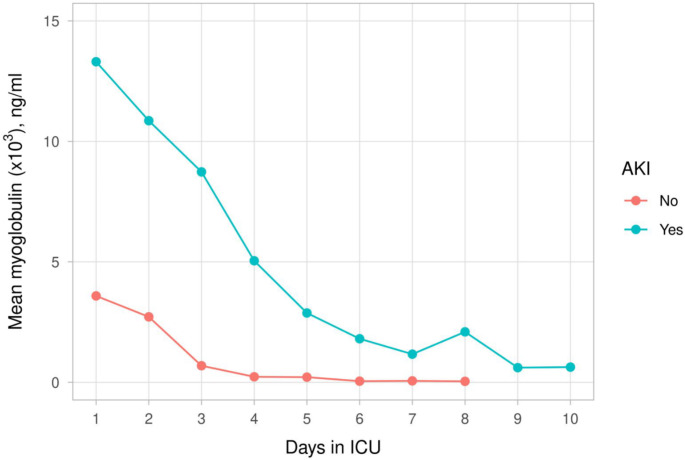
Mean serum myoglobin levels - first 10 days of follow-up with and without AKI

## Discussion

A retrospective analysis of ICU follow-up of earthquake victims revealed a high rate of acute kidney injury. Most of these patients received renal replacement therapy. A total of 77% patients underwent surgical procedures, with the majority (36%) requiring extremities surgery, including fasciotomy, due to compartment syndrome. Additionally, 24% patients underwent extremity amputation. The mNUTRIC score, serum BUN and blood lactate level were identified as independent risk factors for 28-day mortality in a Cox regression model.

Patients who have been affected by major natural disasters, such as earthquakes, should be treated in well-equipped health centers staffed by multidisciplinary teams. In some cases, there may not be any multidisciplinary hospitals around the earthquake, or the earthquake may affect the area in which hospitals are available. In 1999, the most developed region of Turkey was hit by a devastating earthquake in the Marmara region. A medical unit of the Medical Corps of the Israeli Defense Forces providing intensive care in the setting of a field hospital, deployed to the site of a major urban disaster [12]. The 12-bed intensive care unit was run by the unit. It was staffed by three doctors and eight nurses/paramedics. The patient mix was a total of 63 patients, including five major trauma, 20 acute cardiac, 15 various acute illnesses and 11 surgical and post-operative patients. The 6 February 2023 earthquake in Turkey, which occurred in the south-eastern region, had a minimal impact on the country’s health system. Those who were injured in the earthquake were transported to hospitals across the country, with the majority being treated in nearby cities. The city of Kayseri borders on the city of Kahramanmaras, the epicenter of the earthquake. The results of this analysis show that patients with crush injuries underwent many surgical procedures. A large proportion of patients underwent RRT, and some were placed on mechanical ventilators. Although the earthquake was felt, the health system was not affected. Patients from the earthquake zone were successfully treated.

A retrospective analysis of patients who had been affected by the 1999 Marmara earthquake, which occurred 24 years prior to the Kahramanmaraș earthquake, revealed that (13 patients) 72% of those who were under intensive care for renal failure ultimately required haemodialysis, as reported by Demirkıran et al. [5]. In a study of the cases of the Great Marmara earthquake, Kazancıoğlu et al. examined 60 patients diagnosed with crush syndrome and found that 40 patients (66%) had developed renal failure necessitating dialysis [13]. In 2008, 32 ICU patients affected by the Wuhan earthquake in China were evaluated. Of these patients, 53% developed crush injury syndrome and 34% developed acute renal failure. A total of 18% of these patients died [4]. Similar to our patients, a high rate of renal damage is observed in patients with crush injury, as seen in all of the above studies.

Organ amputations, especially of the lower limbs, are a significant condition that affects both the ICU and the life of the victims after the ICU. Analyses of patient data following major earthquakes around the world have shown that over 20% of organs were amputated [5, 14, 15]. The study found a 24% rate of limb amputation. High APACHE II and SOFA scores were found to be associated with increased risk of amputation. In the above analyses, it is noted that patients had multiorgan failure same as our study. The initial APACHE 2 score and the subsequent SOFA score are utilized in the management of ICU patients in daily practice as two critical parameters that indicate the severity and mortality of the disease [16, 17].

The modified NUTRIC score, a tool designed for nutritional assessment in intensive care units, encompasses sub-parameters that signal the severity of critical illness [18]. Consequently, it can also be regarded as a disease severity scoring system [19]. In the context of survival analysis and logistic regression, the elevated mortality rate observed in patients with high modified NUTRIC scores can be interpreted in this manner.

The health facilities in the region where the earthquake occurred may be important in assessing the in-hospital mortality of people affected by the earthquake [20, 21, 22, 23]. The mortality rate for these patients is over 50%. Mortality rates are higher in patients with crush syndrome, need for renal replacement therapy, shock and need for multiple surgeries [4, 21, 24]. In this analysis, the presence of malnutrition and high levels of BUN and lactate in the blood were found to be independent risk factors for 28-day mortality. The relationship between malnutrition and mortality in earthquake patients requiring intensive care has not been studied. In this study, the relationship between the mNUTRIC score and mortality was determined for the first time. A study of patients affected by the Kahramanmaraş earthquake but taken to hospitals in another city, Adana, found higher blood BUN and lactate levels in patients who died [24].

This study has some limitations. There is no information on how long the patients were under the collapsed building. But all these patients were treated within the first 48 h after the earthquake. The other limitation is the relatively small number of patients. These results cannot be generalized.

## Conclusion

On 6 February 2023, two major earthquakes occurred in the province of Kahramanmaras, Türkiye. In this study, critically crush injury patients requiring adult ICU were examined; Acute kidney injury occurred in 65% of patients. Most of these patients required renal replacement therapy. More than 75% of patients underwent surgery. An amputation of a limb was carried out in 24% of the victims. Patients died in 13% of cases. High mNUTRIC score, high blood BUN and lactate levels were independent risk factors for28-day mortality. These results suggest that patients rescued from the earthquake should be treated in hospitals with multidisciplinary health teams. It is imperative that operating theatre rooms in hospitals and certain specialist departments, such as orthopedics, vascular surgery, neurosurgery and general surgery, are equipped to respond to patients in the event of a disaster.

## Electronic supplementary material

Below is the link to the electronic supplementary material.

Supplementary Material 1

## Data Availability

No datasets were generated or analysed during the current study.
