# Supplementary material for: Retrospective analysis of earthquake related crush injurie patients in ICU: 6-February earthquake in Türkiye
Source: Eur J Trauma Emerg Surg. 2025 Feb 21;51(1):116. doi: 10.1007/s00068-025-02771-4 (PMC11845545; doi:10.1007/s00068-025-02771-4)
Supplement: Supplementary file 1 — Supplementary Material 1 [file 68_2025_2771_MOESM1_ESM.pdf]

Supplemental table 1. Serum creatinine levels for first 10 days in all patients, patients with and without AKI in ICU

**Creatinin, mg/dl**	**All patients Mean±SD**	**AKI (+) Mean±SD**	**AKI (-) Mean±SD**	**P**
Day 1	2.40±1,98	2.95±1.99	1.06±1.19	< 0.001
Day 2	1.92±1.70	2,34±1.72	0.90±1.16	0.003
Day 3	1.72±1.57	2,20±1.59	0.76±1.01	< 0.001
Day 4	1,69±1.57	2,26±1.59	0.63±0.83	< 0.001
Day 5	30.86±179.46	43.28±212.79	0.38±0.21	0.511
Day 6	1.98±1.81	2.42±1.80	0.38±0.22	< 0.001
Day 7	2.17±1.87	2.64±1.81	0.32±0.13	< 0.001
Day 8	2.30±1.87	2.80±1.77	0.34±0.17	0.014
Day 9	2.47±2.04	3.22±1.87	0.40±0.19	0.012
Day 10	2.39±2.13	3.26±1.96	0.37±0.21	0.039

Supplemental table 2. Serum CK levels for first 10 days in all patients, patients with and without AKI in ICU

**CK, μ/L**	**All patients Mean±SD**	**AKI (+) Mean±SD**	**AKI (-) Mean±SD**	**P**
Day 1	38098.63±53074.37	47754.92±59756.89	15567.27±19905.58	**0.003**
Day 2	26417.31±49218.75	32801.36±56832.59	11095.60±14850.94	0.153
Day 3	21964.42±51213.40	28724.16±60544.30	6996.42±8238.39	0.191
Day 4	12222.58±19242.86	16242.84±22494.12	5254.13±8507.36	0.078
Day 5	17994.69±60279.83	22925.00±68819.68	2587.50±3830.76	0.415
Day 6	17917.70±50934.24	22245.05±56740.97	1473.80±1529.49	0.429
Day 7	10559.08±25461.05	13167.66±28382.04	1168.20±1026.52	0.363
Day 8	7815.57±20044.45	9768.60±22296.03	491.75±479.47	0.426
Day 9	4985.44±9813.84	5495.50±10363.12	905.00	0.689
Day 10	3557.56±6007.07	4706.33±7224.49	1260.00±1406.87	0.454

Supplemental table 3. Serum myoglobuline levels for first 10 days in all patients, patients with and without AKI in ICU

**Myoglobulin, ng/mL**	**All patients Mean±SD**	**AKI (+) Mean±SD**	**AKI (-) Mean±SD**	**P**
Day 1	11259.51±14573.27	14842.72±15969.54	2500.55±2316.23	**0.002**
Day 2	8687.80±11399.22	9861.53±11844.89	1058.50±1119.35	0.327
Day 3	7495.76±16886.62	9600.80±18941.23	479.00±687.97	0.436
Day 4	3440.04±7479.08	4659.78±8568.35	268.74±272.36	0.277
Day 5	2263.34±4899.83	2821.25±5521.79	403.66±317.05	0.478
Day 6	1417.28±3335.42	1766.71±3766.23	194.30±217.36	0.591
Day 7	1010.40±2097.91	1010.40±2097.91	-	-
Day 8	1273.00±2047.50	1585.25±2222.53	24.00	0.574
Day 9	610.00±213.02	610.00±213.02	-	-
Day 10	632.00	632.00	-	-
